# The Sago Trees

**Published:** 1882-01

**Authors:** 


### THE SAGO TREES.

IT SEEMS a little curious to eat the solid trunk of a tree, but there is a tree in the East Indies which makes an agreeable and wholesome food for thousands of people. The food is well known in this country, though the tree itself is never seen, being the sago so often made into puddings and custards. A full-grown tree is cut down close to the ground. A strip of the bark is then torn off, laying bare the pith, which is about as soft as dried apples.

With a club of heavy wood, pointed at the end with sharp quartz rock, the natives cut out this pith, which is carried to the water-side, and, being mixed with water, is kneaded and pressed against a strainer until the starch is dissolved and passed through the strainer.

The water holding the starch in solution is then passed through the trough, where the sediment is deposited and the water is drawn off. It is then put up in cylindrical cakes of about thirty-six pounds weight and sold as raw sago.

The raw sago, to prepare it for use, is broken up, dried by exposure to the sun, powdered and sifted. This flour is made into cakes, easily baked, which are very delicious if eaten with butter and a mixture of sugar and grated cocoanut.

The cakes are not only eaten hot, but are often dried in the sun and put away in bundles for future use. They will keep good for years, it is said. Children are fond of them, even when hard and dry; but older persons generally dip them in water and toast them, when they relish as well as when fresh baked, or by soaking and boiling, make them serve as puddings, or in the place of vegetables.

This food, as may be imagined, is extrordinarily cheap, costing much less than rice among the Hindoos, or potatoes among the Irish.

A good-sized trunk of a sago tree twenty feet long and five in circumference will make at least thirty bundles of thirty pounds each. Each bundle, it is computed, will make sixty cakes, allowing three cakes to a pound, and five cakes are considered by the natives sufficient for a full day's food. A single good-sized tree will, therefore, furnish food for a native for an entire year, and many of them live upon it almost exclusively.

One needs to labor only a few days to secure this supply of food for the year. A man can reduce a tree to powder in ten days, and a woman, in the same time, can reduce it all into cakes. By steady labor for twenty days, therefore, provision may be laid up for a year.

But such cheap living proves favorable neither to health of body nor of mind. A uniform diet of sago, varied only by fish, rarely by fruit or vegetables, is not good for the body, and the want of stimulus to exertion is prejudicial to the character.

What is got easily is generally worth little; and the natives, having no occasion for physical toil or for careful thrift, have no force of character.

